# Distributions of α- and δ-TOCopherol in Intact Olive and Soybean Oil-in-Water Emulsions at Various Acidities: A Test of the Sensitivity of the Pseudophase Kinetic Model

**DOI:** 10.3390/antiox11122477

**Published:** 2022-12-16

**Authors:** Lucía Fernández-Ventoso, Artai Toba-Pérez, Sonia Losada-Barreiro, Fátima Paiva-Martins, Carlos Bravo-Díaz

**Affiliations:** 1 Departamento Química-Física, Facultad de Química, Universidade de Vigo, 36310 Vigo, Spain; 2 REQUIMTE-LAQV, Departamento de Química e Bioquímica, Faculdade de Ciências, Universidade do Porto, 4169-007 Porto, Portugal

**Keywords:** tocopherol, antioxidants, emulsions, pseudophase kinetic model, distribution

## Abstract

During the last years, the formalism of the pseudophase kinetic model (PKM) has been successfully applied to determine the distributions of antioxidants and their effective interfacial concentrations, and to assess the relative importance of emulsion and antioxidant properties (oil and surfactant nature, temperature, acidity, chemical structure, hydrophilic-liphophilic balance (HLB), etc.) on their efficiency in intact lipid-based emulsions. The PKM permits separating the contributions of the medium and of the concentration to the overall rate of the reaction. In this paper, we report the results of a specifically designed experiment to further test the suitability of the PKM to evaluate the distributions of antioxidants among the various regions of intact lipid-based emulsions and provide insights into their chemical reactivity in multiphasic systems. For this purpose, we employed the antioxidants α- and δ-TOCopherol (α- and δ-TOC, respectively) and determined, at different acidities well below their p*K*a, the interfacial rate constants *k_I_* for the reaction between 16-ArN_2_^+^ and α- and δ-TOC, and the antioxidant distributions in intact emulsions prepared with olive and soybean oils. Results show that the effective interfacial concentration of δ-TOC is higher than that of α-TOC in 1:9 (*v*/*v*) soybean and 1:9 olive oil emulsions. The effective interfacial concentrations of tocopherols are much higher (15-96-fold) than the stoichiometric concentrations, as the effective interfacial concentrations of both δ-TOC and α-TOC in soybean oil emulsions are higher (2-fold) than those in olive oil emulsions. Overall, the results demonstrate that the PKM grants an effective separation of the medium and concentration effects, demonstrating that the PKM constitutes a powerful non-destructive tool to determine antioxidant concentrations in intact emulsions and to assess the effects of various factors affecting them.

## 1. Introduction

Because unsaturated lipids are prone to oxidation, one of the major challenges for the food industry is to maintain the quality of lipid-based products during processing and storage until use by consumers [1,2,3]. The most characteristic change that lipid-based food material suffers is probably the production of unpleasant smells and the development of unpleasant tastes, usually accompanied by changes in the color of the food, the loss of organoleptic properties, and a significant decrease in the quality of the food (e.g., loss of essential fatty acids, degradation of vitamins and pro-vitamins, formation of harmful and odor-intensive compounds, etc.) [4,5]. As a result, the oxidative process leads to the formation of a wide range of volatile and non-volatile degradation products, with rancidity as the main effect. To extend food shelf life and to reduce food waste in as much as possible, food chemists aim to control and minimize the oxidative process [6,7,8].

Lipids in foods are mostly found as an oil-in-water emulsion, which is a multiphasic system where surfactants create an interfacial region between the continuous and dispersed phases [7,9]. Each region behaves as a microreactor, where compounds may concentrate or dilute in each region depending on their chemical nature and on the nature of the emulsion components [6]. Understanding how the properties and composition of the interfacial region affects the chemistry of lipid oxidation is very important and has led to new strategies to inhibit oxidation, such as targeting antioxidants (AOs) to the interfaces to increase their concentration (and hence their efficiency) [10,11], creating charged surfaces that repel metals, [12] adsorbing solid particles to create pickering emulsions [13,14,15], or even creating thick interfaces to limit lipid-prooxidant interactions. As food formulations continue to employ polyunsaturated fatty acids, the development of new antioxidant strategies is necessary, focusing the efforts of many researchers.

### 1.1. The Problem of Determining Antioxidant Distributions in Intact Emulsions

Not long ago, interpreting the inhibition of lipid oxidation processes between antioxidants and peroxyl radicals in lipid-based emulsions was intractable [16,17,18,19]. Part of the problem lied in the lack of methods allowing separation and quantification of the contributions of the distribution of reactants and of the medium effects to the total rate of inhibition [20,21,22]. Particularly, knowing the distributions of antioxidants in emulsions was challenging for a long time, preventing a full rationalization of their effects because emulsions have regions with different solvent properties that affect both the reactivity and concentrations of antioxidants. This objective was (and still is) one of the most important challenges of food chemists, encouraging them to make huge and imaginative efforts to develop methods to determine the effective concentrations of antioxidants in the various regions of the emulsions [7,23,24,25].

**Scheme 1:**
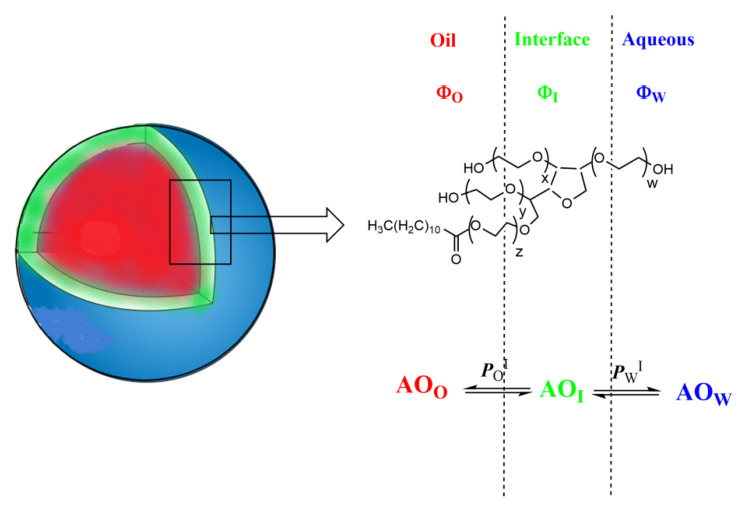
Illustrative representation of an emulsion droplet (**left**) and the conceptual division in the oil, interfacial, and aqueous regions (**right**), showing the distribution of an antioxidant AO between the oil (O), interfacial (I), and aqueous (W) regions, characterized by the partition constants between the oil-interfacial, *P*_O_^I^, regions and those between the aqueous-interfacial, *P*_w_^I^, regions.

Attempts to determine quantitatively the distributions of antioxidants in emulsions were first made on the basis of the published values of the partition constants, *P*_w_^O^, between the oils and water [26,27,28,29]. The efforts were, unfortunately, quite unsuccessful because in emulsions, antioxidants distribute between the oil, interfacial, and aqueous regions, Scheme 1. Consequently, to describe their partitioning, the partition constants *P*_O_^I^ (that between the oil-interfacial regions) and *P*_w_^I^ (that between the aqueous-interfacial regions) are necessary. *P*_w_^I^ and *P*_O_^I^ are defined according to Equations (1) and (2), where parentheses denote the effective concentrations of the AO in each region (moles per liter of the particular region). It is worth noting that in the limit case, where no surfactant is added, i.e., in a binary oil-water system, the ratio of *P*_O_^I^ and *P*_w_^I^ equals that in the binary system (Equation (3)) [21,30]. Therefore, only the ratio of *P*_O_^I^/*P*_w_^I^ can be calculated from *P*_w_^O^ values, and the individual values cannot be determined [7,31].

In contrast, the oil-water partition constants’ *P*_w_^OIL^ values are rather scarce in the literature, probably because their determination may be difficult and time-consuming, making some researchers assume, erroneously and quite often, that the *P*_w_^OIL^ values are proportional (or similar) to the much more readily available values of the octanol-water, *P*_W_^OCT^, partition constants [32,33,34]. These *P*_W_^OCT^ values can be easily estimated by employing a variety of computational methods, such as acd Log *p*, Molinspiration, PubChem, XlogP3, Mlog *p*, Chem Draw log *p*, etc. These software packages are readily available and offer limited free web access; however, they do not provide the same values for the same molecules because the algorithms and approximations they employ to compute molecular properties are not the same [32,35]. Nonetheless, the use of *P*_W_^OCT^ to predict the distribution of antioxidants in binary oil-water systems is not reliable at all because the properties of the solvents (octanol and the food oil) are quite different, making the experimental *P*_w_^OIL^ values completely different from computer-estimated *P*_w_^OCT^ values, which frequently leads to the misperception of experimental results [6].

(1) $$P_{W}^{I}=\frac{{(\mathrm{AO}}_{I})}{{(\mathrm{AO}}_{W})}$$

(2) $$P_{O}^{I}=\frac{{(\mathrm{AO}}_{I})}{{(\mathrm{AO}}_{O})}$$

(3) $$P_{W}^{O}=\frac{{\left(\mathrm{AO}\right)}_{O}}{{\left(\mathrm{AO}\right)}_{W}}=\frac{P_{W}^{I}}{P_{O}^{I}}$$

Further attempts to determine the distributions of AOs in emulsions were made by analyzing the content of antioxidants in each of the resulting mixtures after breaking down the emulsions [36,37]. Classical methods to determine the distribution of molecules in binary systems cannot be employed in emulsified systems [36,38,39]. Unfortunately, the existing equilibria in the emulsions are disrupted upon breaking down the emulsions; therefore, the results obtained were biased and of limited value.

### 1.2. Pseudophase Kinetic Approach: Determining Antioxidant Distributions in Intact Emulsions

Some of the experimental issues in estimating the effective concentrations of antioxidants were overwhelmed with the application of pseudophase kinetic methods [7,21,30,31]. The basic idea underlying such approach is the use of a chemical probe that is sensitive to the antioxidant, reacting with it at rates that are proportional to the interfacial concentration of the antioxidant and, therefore, responsive to its distribution in the intact emulsion. Thus, the chemical probe 4-hexadecylbenzediazonium ion, 16-ArN_2_^+^ was employed due to it reactions with virtually all antioxidants and, most importantly, the fact that its reactive –N_2_^+^ group is distributed exclusively in the interfacial region of emulsions. Details can be found elsewhere [7,21,30,31] and in references therein.

Application of the pseudophase kinetic model to different emulsions under different experimental conditions provided, for the first time, reasonable estimates of (1) the partition constants *P*_O_^I^ and *P*_W_^I^ for the distributions of antioxidants, which are obtained in the intact emulsions, and (2) the intrinsic rate constant, *k*_I_, for the reaction between the chemical probe and the antioxidants, which takes place exclusively in the interfacial region of the emulsion. The partition constants *P*_O_^I^ and *P*_W_^I^ values are then used to calculate the percentage of antioxidants in each region of the emulsion, as well as the effective concentrations of the AOs in those regions. The model also provided natural explanations, based on molecular properties, of the effects of a variety of parameters (HLB of the surfactant, acidity, temperature, nature and type of the oil, etc.) on the distribution of antioxidants between the regions of the various emulsions and nanoemulsions employed [6,7,10,35,40].

### 1.3. Separating Medium from Concentration Effects on the Rates of Reactions in Emulsions: Is the Pseudophase Kinetic Model a Trustable Method?

During the reviewing process of the various papers we have written on the application of pseudophase models to determine the distributions of AOs in intact lipid-based emulsions, we received a number of similar critical questions. One of the most frequent was focused on the sensitivity of the model for effectively separating both contributions (medium and concentration) to the overall rate of the reaction, which caused reviewers to (reasonably) wonder if the obtained values of *k*_I,_ *P*_O_^I^, and *P*_W_^I^ could be somewhat biased because of mathematical or experimental artefacts. In other words, the reviewers wondered if the pseudophase kinetic model and the associated experimental methodology were sensitive enough to separate the contributions of the concentration and the medium to the overall rate constant effectively.

We feel that it can be instructive to provide an answer based on physical evidence supported by experimental results, despite having obtained self-consistent results, and that pseudophase models have been employed for years to successfully interpret chemical reactivity in colloidal systems [21,41,42,43,44,45,46]. Bearing this in mind, we analyzed the problem on the basis of a specifically designed experiment aimed to provide further support to the operative assumptions of the pseudophase model.

**Scheme 2:**
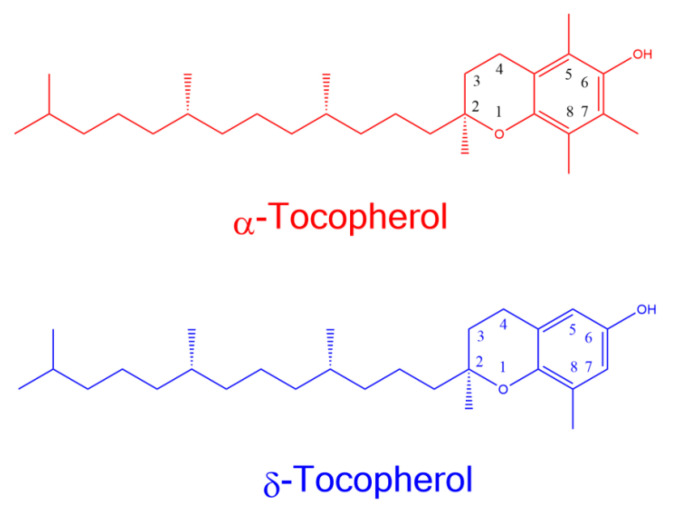
Chemical structures of two of the four major forms of vitamin E (α-, β-, γ-, and δ-TOCopherols). In α-TOCopherol (the most biologically active form), the chromanol ring is fully methylated, whereas δ-TOCopherol is only methylated in position 8. Both tocopherols have a saturated phytyl side chain attached at C2 and have three chiral centers.

Our rationale in designing the experiment was as follows. The antioxidants α- and δ-TOCopherol ((*R*)-2,5,7,8-tetramethyl- 2-((4′*R*,8′*R*)-(4′,8′,12′-trimethyltridecyl)chroman-6-ol and (*R*)-2,8-dimethyl-2-(4′*R*,8′*R*)-4′,8′,12′,-trimethyltridecyl)chroman-6-ol, respectively), whose chemical structures are shown in Scheme 2, were chosen because of the similarity in their chemical structures (in addition to their being relevant natural antioxidants). The difference between them is the degree of methylation of the chromanol ring: α-TOCopherol is methylated in the 5, 7, and 8 positions on the chromanol ring, whereas δ-TOCopherol is only methylated in the 8 position. Thus, both antioxidants should have a similar hydrophobicity. The mechanism of the reaction between 16-ArN_2_^+^ and α-TOC is different from that with δ-TOCopherol because of the differential degree of methylation in the chromanol ring. However, in both cases, the rate of the reaction depends inversely on the acidity of the solution, i.e., *k*_I_ α 1/[H^+^], such that the variation of the logarithm of the intrinsic rate constant, log *k*_I_, with pH is linear.

### 1.4. Applicability and Importance of the Study

We, thus, applied the methodology of the pseudophase kinetic model and determined their distribution and the intrinsic rate constants *k_I_* in olive and soybean oil-in-water emulsions at different acidities (pH = 3–6) well below the p*K*_a_ of the antioxidants (p*K*_a_ = 9–10) in order to minimize any potential ionization of the antioxidants, which eventually could alter their partitioning. Note that the ionized forms of the antioxidants are much more soluble in the aqueous phase than the neutral forms. If the separation of medium and concentration effects is effective when applying the PKM, we would expect to obtain similar partition constants for α-TOC and δ-TOC independently of the acidity of the medium both in olive and soybean emulsions. However, we should obtain different rate constants that, according to the reaction mechanisms, should increase upon lowering the acidity. We have also used the PKM to undertake a study on the distribution of α-TOC and δ-TOC in both emulsions because a literature survey indicated that little is known about the distribution of α-TOCopherol in oil-in-water emulsions, and even less about that of δ-TOCopherol, despite both antioxidants having been employed for years as natural antioxidants in foods and cosmetics [47,48].

In the present paper, we evaluated the effects of the surfactant concentration on their distributions and effective concentrations in the intact olive and soybean oil emulsions. The study is relevant because we have recently demonstrated that there is a direct relationship between the interfacial concentrations of antioxidants and their efficiency in minimizing lipid peroxidation. The antioxidant properties of the tocopherols arise from their ability to scavenge free radicals as a result of the reactive –OH group on their phenolic ring, which can undergo hydrogen transfer reactions, reacting with different free radicals generated during the oxidation processes (e.g., alkoxyl, peroxyl and other C-centered radicals) and converting them into stable non-radical products, along with forming a tocopheryl radical. Tocopheryl radicals are relatively long-lived and do not react with other lipid substrates; instead, they can react with other peroxyl radicals to form stable tocopherol quinones [49]. Thus, a single tocopherol molecule can react with two radicals and inhibit the propagation step of the free-radical autoxidation pathway [50,51,52]. The reactivity order reported for the hydrogen-removing ability of tocopherols is highest for α- > β- > γ- > δ- tocopherol, according to the differences in their bond dissociation energies [51,53]. Alkoxyl radicals might be important in that they can be generated by the decomposition of alkyl peroxides (ROOH) induced by heat, radiation, or a reaction with transition metal ions, or from preexisting hydroperoxides in the oil.

As we will see, the results obtained demonstrate the sensitivity (and utility) of the pseudophase model in determining the distributions of antioxidants in emulsions. Indeed, the results obtained demonstrate that pseudophase kinetic analyses provide a unique, versatile, and robust solution for interpreting chemical reactivity in emulsions, separating the medium from the concentration effects. The medium effects reflect the solvent properties of a reaction region, and the distributions of reactants depend on their solubilities in each region. In addition, the approach permits identifying the relative importance of various parameters affecting the distribution of antioxidants in emulsions (such as oil hydrophobicity, emulsifier structure and HLB, temperature, and acidity) in reactant distributions. The pseudophase kinetic model offers, therefore, a new and unique method for determining the distributions of antioxidants in intact emulsions, allowing analysis of the effects of various parameters on their efficiency and, eventually, identifying the most efficient antioxidant for a particular food application.

## 2. A Brief Insight into the Chemistry of Aryldiazonium Ions: C- and O-Coupling Reactions

Aryldiazonium ions belong to an important class of organic compounds that have been recognized as one of the most versatile and valuable reagents in organic synthesis for more than 150 years, and they are currently employed, among others, in the functionalization of surfaces in order to probe interfacial compositions of micelles, microemulsions, reverse micelles, and cyclodextrins and to determine the distribution of antioxidants in intact lipid-based emulsions.

**Scheme 3:**
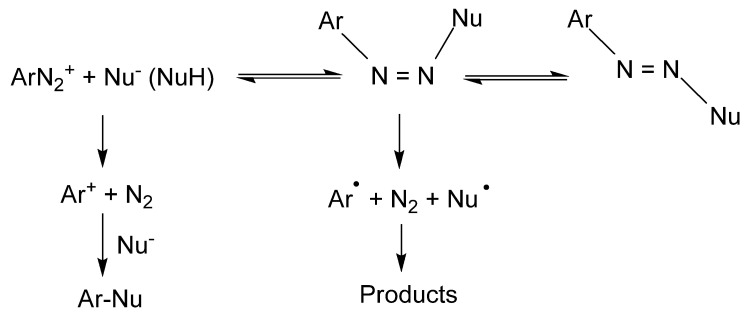
Nucleophilic addition mechanism forming Ar-N=N-Nu adducts in the (*Z*)- and (*E*)-configurations. The competitive spontaneous decomposition of ArN_2_^+^ is also included. Adapted from ref. [54] with permission, Copyright J. Wiley & Sons.

Aryldiazonium, ArN_2_^+^, ions may function as Lewis acids, reacting with nucleophiles (Lewis bases, Nu^–^ or NuH followed by loss of a proton) to give covalently bonded adducts, ArN_2_-Nu, at the β-nitrogen of the arenediazonium ion, which is the electrophilic reactive center, Scheme 3 [54].

**Scheme 4:**
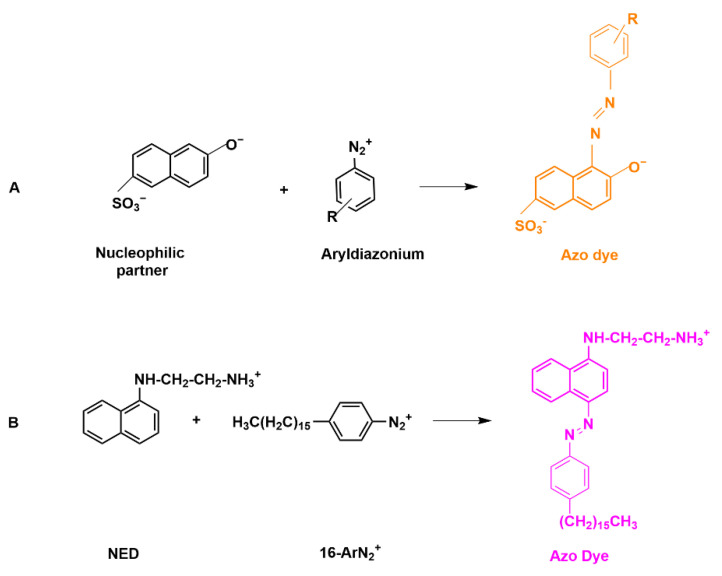
Representative coupling reaction leading to the formation of azo dyes between ArN_2_^+^ and (**A**) the Na salt of 2-naphthol-6-sulfonic acid (nucleophilic partner), (**B**) the coupling agent N-(1-Naphthyl)ethylenediamine (NED). The latter reaction is exploited to monitor the reaction between the chemical probe 16-ArN_2_^+^ and the antioxidants in intact emulsions (see Section 3.3 below).

Typical examples of covalently bonded adducts are the azo dyes, which are formed through C-coupling reactions when ArN_2_^+^ reacts with aromatic substrates containing hydroxyl (e.g., tocopherols) or amino groups, Scheme 3 [55]. C-coupling reactions mostly take place at the *o*- and *p*-positions, and, in fact, *m*-substitutions have never been observed [55,56]. This is the case of δ-TOC, which has the positions 5 and 7 of the chromanol ring available, Scheme 2. Therefore, the reaction between 16-ArN_2_^+^ and δ-TOC is expected to take place through the electrophilic aromatic substitution mechanism, obtaining Wheland intermediates [57] with subsequent loss of a proton in an irreversible step [58]. Phenols and naphthols react through their ionized forms (phenoxide or naphthoxide ions), Scheme 4, at rates as much as 10^10^ times faster than with the non-ionized forms. Hence, the rates of C-coupling reactions show an inverse dependence with [H^+^], that is, the logarithm of the rate constant, log *k*, with pH is linear with a slope close to the unit [55,59,60].

**Scheme 5:**
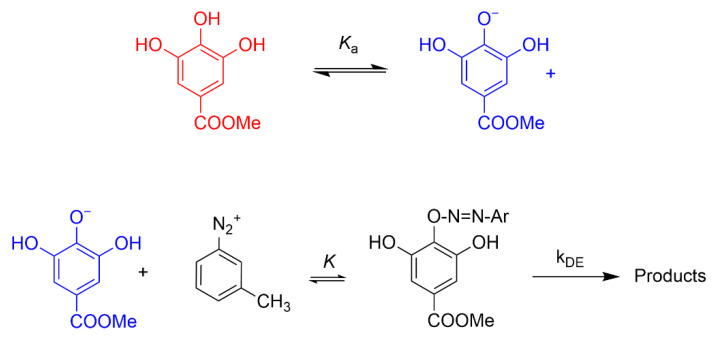
Proposed mechanisms for the reaction between ArN_2_^+^ and methyl gallate. Similar mechanisms have been proposed for reactions of ArN_2_^+^ with antioxidants bearing catechol moieties [63,64]. Adapted from ref. [54] with permission, Copyright J. Wiley & Sons.

When the *o*- or *p*-positions of the aromatic ring are blocked, such as in α-TOCopherol, C-coupling reactions are not possible, ArN_2_^+^ undergoes O-coupling reactions, leading to the formation of O-adducts, Scheme 5. The leaving ability of the nucleophile Nu^–^ involved in the reaction has a strong influence on their stability, such that if the nucleophile Nu^–^ is a poor leaving group (e.g., the ascorbate ions), some stabilization may take place by conversion to a thermodynamically stable isomer (e.g., *Z-E* isomerization) [61,62]; otherwise, they may split homolitically, obtaining reduction products.

When two hydroxyl groups are present in the aromatic ring, the reactivity of the phenol increases, but the effects are not additive and are clearly depend on the relative position of the –OH groups in the benzene ring [57,60,65]. For instance, resorcinol (1,3-C_6_H_4_(OH)_2_) contains two nucleophilic centers able to couple, with the dianion coupling more than 10^4^ times faster than that of the monoanion [66]. In contrast, catechol (1,2-C_6_H_4_(OH)_2_), hydroquinone (1,4-C_6_H_4_(OH)_2_), and t-butylhydroquinone are oxidized by ArN_2_^+^ ions because isomerization is not possible, and the adduct splits homolitically to finally give reduction products [67,68,69]. Reactions involving trihydric phenols (e.g., gallic acid and its derivative methyl gallate) have been recently studied [63,64,70]. Because the reacting species is the phenolate ion in all cases, the rate of the reaction shows the same inverse dependence with [H^+^] as that of C-coupling reactions.

## 3. Materials and Methods

### 3.1. Materials

All chemicals were of the highest purity available (>90%) and used as received. All aqueous solutions were prepared by employing deionized water (conductivity < 0.1 mS cm^−1^). Polyoxyehthylene sorbitan monolaurate (Tween 20, ρ = 1.11 g/mL) and the coupling agent N-(1-naphthyl) ethylenediamine (NED) were purchased from Acros Organics. The coupling agent NED and the antioxidants (+)-δ-TOCoferol (90%, MW = 402.7 g/mol) and (±)-α-TOCoferol (96%, MW = 430.72 g/mol) were from Sigma-Aldrich (St. Louis, MO, USA).

The soybean and olive oils were purchased from a local supplier and stripped from their endogenous antioxidants by washing them with a 0.5 M NaOH solution and passing them twice through an activated Al_2_O_3_ column. The absence of endogenous antioxidants was checked by HPLC, according to standard procedures (IUPAC method 2.432). 4-Hexadecylbenzenediazonium tetrafluoroborate, 16-ArN_2_BF_4_, was prepared from 4-hexadecylaniline (Aldrich, 97%) by diazotization with butyl nitrite in acidic solution, as described elsewhere [30].

### 3.2. Preparation of Emulsions

Oil-in-water emulsions (1:9 *v*:*v*, V_T_ = 10 mL) were prepared, as in previous works, by employing the stripped oils, buffered aqueous solution (0.04 M citric/citrate buffer, pH 3.65), and Tween 20 (0.5–4% *w*/*w*) [6,40]. The antioxidants were added to the stripped oils before homogenization. The mixtures were stirred for 60 s with the aid of a Polytron PT-1600 homogenizer (speed: 30,000 rpm) at room temperature.

### 3.3. Methods

#### Determining Observed Rate Constant (*k_obs_*) Values for the Reaction between 16-ArN_2_^+^ and the AOs in Intact Emulsions

**Figure 1:**
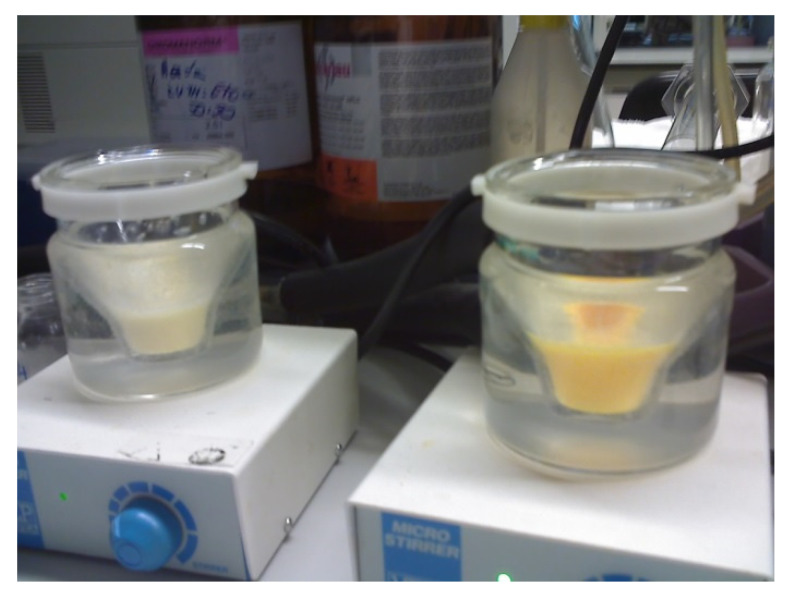
Photographs of the reaction mixtures obtained in olive oil emulsions after reacting 16-ArN_2_^+^ with α-TOC (**left**) and δ-TOC (**right**). The yellowish color is a consequence of the formation of an azo dye between 16-ArN_2_^+^ and δ-TOC.

Figure 1 is representative and shows a photograph taken after completion of the reaction of 16-ArN_2_^+^ with α-TOC (left) and δ-TOC (right) in olive oil-in-water emulsions. The resulting reaction mixture when δ-TOC was employed shows a yellowish color that is not observed in the reaction with α-TOC (left), indicating that different mechanisms are operating (see Section 2).

**Figure 2:**
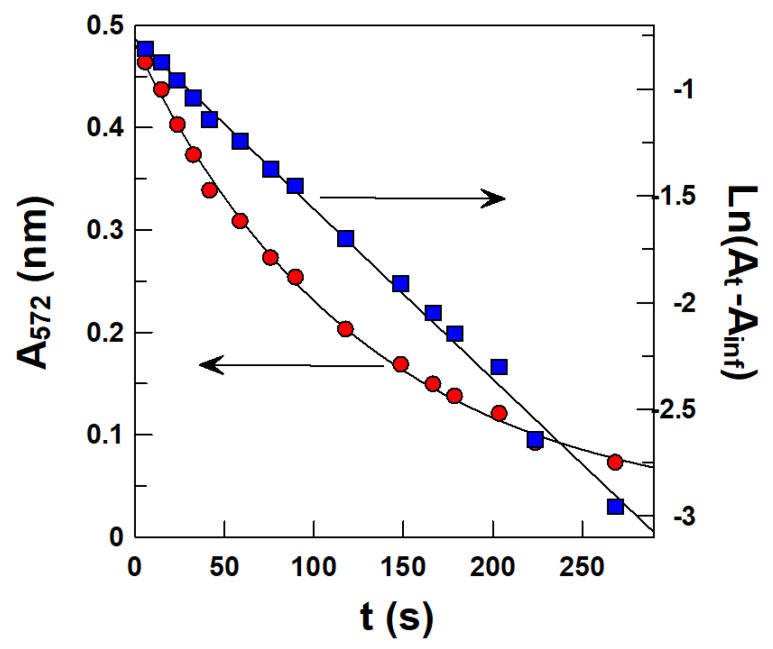
Typical plot showing the changes in absorbance (λ = 572 nm) with time (●) and ln[A_t_ − A_inf_] plot (■). Reaction was carried out in intact 1:9 (*v*:*v*, *o*/*w*) emulsions prepared by employing stripped olive oil, a buffered (pH = 5, citric acid/citrate (0.04 M)) aqueous solution, and Tween 20 (surfactant volume fraction Φ_I_ = V_surf_/V_total_ = 0.04). Experimental conditions: [16-ArN_2_^+^] = 2.90 × 10^−4^ M, [α-TOC] = 4.2 × 10^−3^ M, T = 25 °C.

The reaction between 16-ArN_2_^+^ and antioxidants was monitored spectrometrically using a batch method, as described in detail elsewhere [30,71,72,73]. Because emulsions are opaque, a special protocol, described in detail elsewhere [21,30,74], was employed to monitor reactions in the intact emulsions. Briefly, the protocol exploits the rapid reaction of the chemical probe (16-ArN_2_^+^ ions) with a proper coupling agent, such as N-(1-naphthyl)ethylenediamine, NED, to yield a stable azo dye. The solution is diluted with a 50:50 (*v*:*v*) ethanol:butanol mixture to yield an optically transparent, homogeneous solution, whose absorbance is measured spectrometrically, Figure 2. Reactions were carried out under pseudo first order conditions ([antioxidant] >> [16-ArN_2_^+^]). The coupling agent NED reacts much faster with 16-ArN_2_^+^ than with the antioxidants, such that the reaction of 16-ArN_2_^+^ ions with the antioxidant is effectively quenched. Auxiliary experiments showed that the absorbance of the formed azo dye can be linearly correlated with the concentration of 16-ArN_2_^+^. Details of the procedure can be found elsewhere [6,40].

Values of the observed rate constant, *k_obs_*, were determined by fitting the experimental data to the integrated first order rate (Equation (4), *A_t_*, *A*_inf_, and *A*_0_ stand for the absorbance values at any, infinite, and zero time, respectively). Figure 2 is representative and shows the changes in the absorbance with time and the excellent fit to the integrated and linearized first order equation.

(4) $$\ln\frac{A_{t}-A_{\inf}}{A_{0}-A_{\inf}}=-k_{obs}t$$

### 3.4. Statistical Analysis

Duplicate or triplicate kinetic experiments were run in duplicate or triplicate for 2–3 t_1/2_. The *k_obs_* values were within ±7–9%, with typical correlation coefficients of >0.995. The Dixon’s Q-test was employed in deciding whether to accept or reject the datum before calculating the average of the set of replicates. Data are presented as means ± standard deviation.

## 4. Results and Discussion

### 4.1. Partition Constants P_o_^I^ and Interfacial Rate Constants k_I_ of α- and δ-TOC in Intact Emulsions

**Scheme 6:**
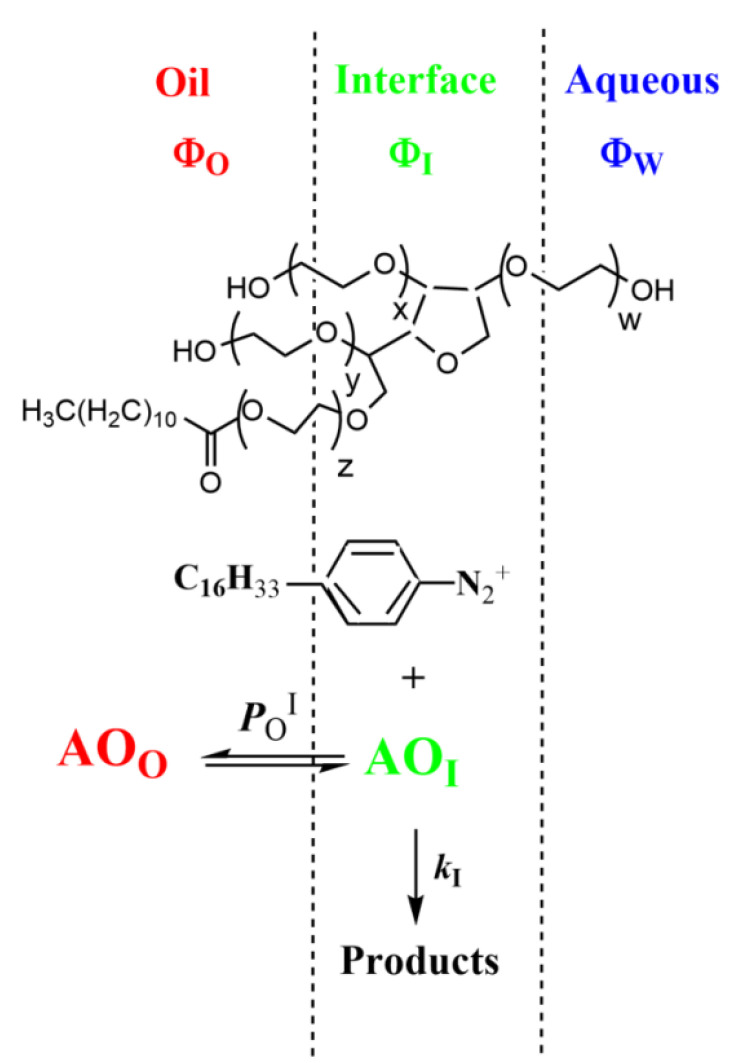
Pictorial representation of the distribution of a water-insoluble antioxidant (e.g., tocopherols) and the location of the chemical probe 16-ArN_2_^+^. *P*_O_^I^ is the partition constant of the antioxidant, *k*_I_ the rate constant between the antioxidant and 16-ArN_2_^+^, and Φ_O_, Φ_I_, Φ_W_ are the volume fractions of the oil (O), interfacial (I), and aqueous (W) regions.

Scheme 6 shows the conceptual basis of the pseudophase kinetic model, which assumes that all components of the emulsion, and particularly the antioxidants (e.g., TOC), are in dynamic equilibrium (that is, the transport of the material between regions is not restricted and is much faster than the undergoing chemical reactions).

TOC is insoluble in water and can only be transferred between the oil and interfacial regions of the emulsion, as illustrated in Scheme 6. Its distribution between the different regions is governed thermodynamically and depends exclusively on its relative solubility in the dispersed and interfacial regions.

By applying the formalism of the pseudophase model (described in detail elsewhere) [21,30,74], Equation (5) can be derived.

(5) $$k_{\mathrm{obs}}=\frac{\left[{\mathrm{AO}}_{T}\right]k_{I}P_{O}^{I}}{\Phi_{{}_{I}}P_{O}^{I}+\Phi_{O}}$$

(6) $$\frac{1}{k_{\mathrm{obs}}}=\frac{\Phi_{O}}{k_{I}\left[{\mathrm{AO}}_{T}\right]P_{O}^{I}}+\frac{1}{k_{I}\left[{\mathrm{AO}}_{T}\right]}\Phi_{I}$$

Equation (5) contains two dependent variables, but only one independent variable. Several approaches are available to solve coupled equations like this, but probably the simpler mathematical treatment is to find a linear relationship between them and fit the experimental data to a linear relationship by means of a least squares fitting procedure. Equation (6) is the reciprocal of Equation (5), and it predicts that plots of 1/*k_obs_* vs. Φ_I_ should be linear with positive intercepts, where the values of the partition constants are obtained from the ratio between the slope and the intercept. Details on the procedure can be found elsewhere [21,30,74].

**Figure 3:**
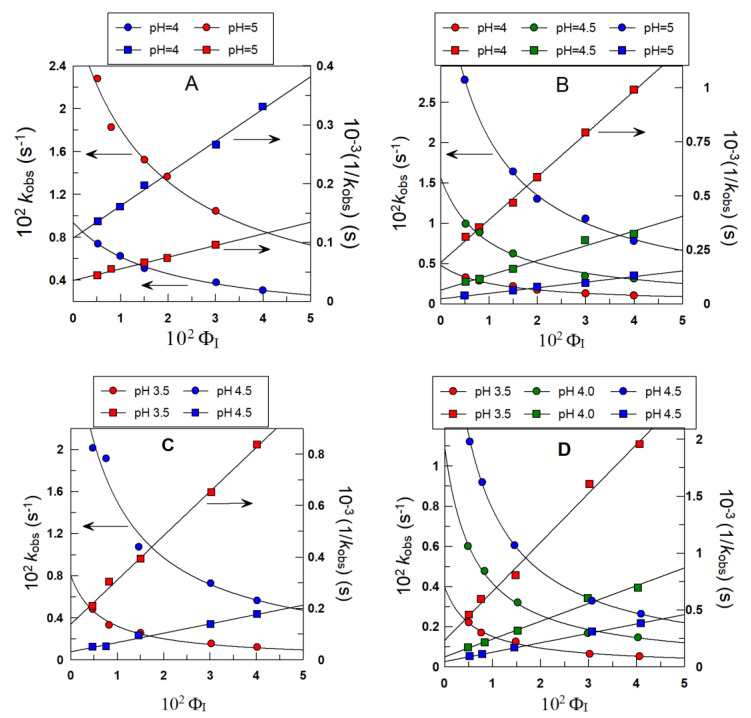
Variations in *k_obs_* with Φ_I_ for the reaction between 16-ArN_2_^+^ and the antioxidants α- (**A**,**C**) and δ-TOC (**B**,**D**) in 1:9 olive (**A**,**B**) and soybean (**C**,**D**) o/w emulsions prepared with buffered aqueous solutions of different acidity. Experimental conditions δ-TOC ([δ-TOC_T_] = 4.0 × 10^−3^ M) y (B) α-TOC ([α-TOC_T_] = 4.0 × 10^−3^ M), T = 25 °C.

Figure 3A–D are illustrative and show the variations of *k_obs_* with Φ_I_ for α- and δ-TOC in 1:9 olive and soybean oil emulsions. Values of the partition constants *P*_O_^I^ and of the rate constants in the interfacial region, *k*_I_, were obtained by employing Equation (6), and the obtained values are displayed in Table 1.

**Figure 4:**
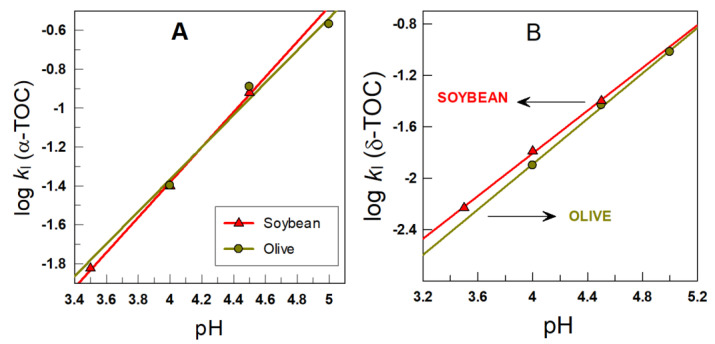
Linear variations of logarithm of the interfacial rate constant *k*_I_ with pH for the reaction between 16−ArN_2_^+^ and α-TOC (**A**) and δ-TOC (**B**) in 1:9 (*o*:*w*) emulsions prepared with olive (●) and soybean (▲) oils.

The results in Table 1 show that the partition constants of δ-TOC are consistently higher than those of α-TOC, independent of the acidity of the aqueous phase, but remain constant upon changing the acidity. In contrast, *k*_I_ values are lower for δ-TOC compared to those of α-TOC, and they increase upon lowering the acidity. The variation of log *k*_I_ with pH is linear for α- and δ-TOC in both emulsions, Figure 4, with slope values ranging 0.8–0.92, very similar to the theoretical ones (slope = 1) that can be expected from the mechanisms of reaction between 16-ArN_2_^+^ and the antioxidants (Section 2).

### 4.2. Distribution and Effective Concentrations of α- and δ-TOC in Soybean and Olive Oil Emulsions

Once the partition constants are known, determining the distribution of the antioxidants and their effective concentrations in the oil (O) and interfacial (I) regions is straightforward by using Equations (7) and (8).

(7) $${\%\mathrm{AO}}_{I}=\frac{{100\Phi }_{I}P_{O}^{I}}{\Phi_{O}{+\Phi }_{I}P_{O}^{I}}$$

(8) $${\%\mathrm{AO}}_{O}=\frac{{100\Phi }_{O}}{\Phi_{O}{+\Phi }_{I}P_{O}^{I}}$$

**Figure 5:**
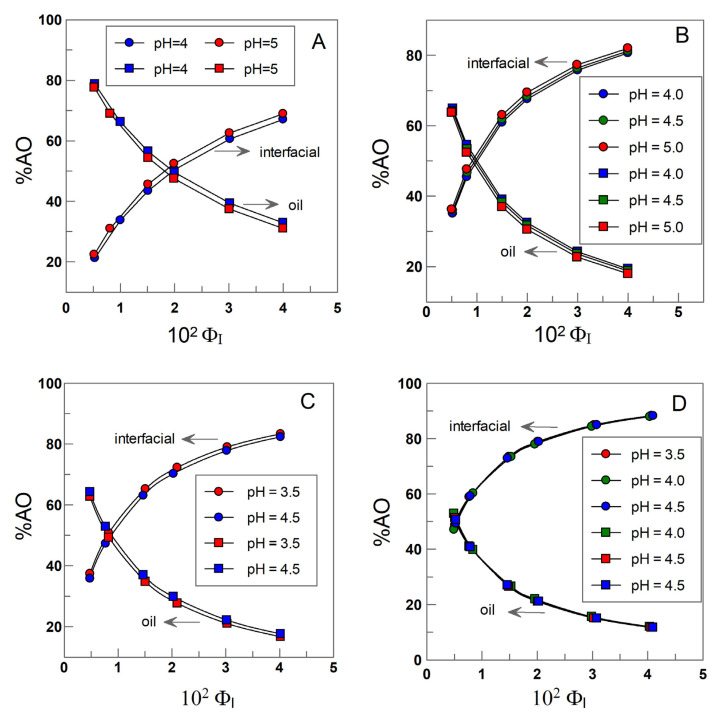
Variation of the percentages of α-TOC (**A**,**C**) and δ-TOC (**B**,**D**) with the surfactant volume fraction Φ_I_ in the oil (■) and interfacial (●) regions of 1:9 olive (**A**,**B**) and soybean (**C**,**D**) oil emulsions at different acidities.

The plots of %AO against Φ_I_ in Figure 5 show the differential distribution of α- and δ-TOC in the emulsions. At any acidity, the distribution of the antioxidants remains unchanged both in olive and soybean oil emulsions because no appreciable ionization takes place in such acidity range. However, the distribution of tocopherols depends on both the chemical structure of the antioxidant and the nature of the oil employed in the preparation of the emulsion.

The results show that at a given pH and Φ_I_ (for example, pH = 4 and Φ_I_ = 0.005), the percentage of δ-TOC in the interfacial region is higher than that of α-TOC in both olive and soybean oil emulsions.

It is worth noting that at the lowest Φ_I_ value employed (Φ_I_ = 0.005), only 20% of α-TOC is located in the interfacial region of olive oil emulsions, with the remaining 80% in the oil region. Note that at the same Φ_I_ values, the percentage of δ-TOC is slightly higher than that of α-TOC. This finding is consistent with a literature report that indicates δ-TOC has a higher polarity of (μ = 1.31) compared to that of α-TOC (μ = 0.87 D).

In both cases, the percentage in the interfacial region increases rapidly with increasing surfactant concentration, such that when Φ_I_ = 0.04, more than 70% of both tocopherols are located in that region. Similar results were found in soybean emulsions, Figure 5C,D.

Note that the percentage of both α- and δ-TOC located in the interfacial region is about 15% higher in soybean oil emulsions than in olive oil emulsions, enhancing soybean oil emulsions’ solubility of TOC in the interfacial region, which can be rationalized on the basis of the higher relative ability of tocopherols to form intra- and intermolecular hydrogen bonds with the polar groups of fatty acids present in soybean oil.

### 4.3. Effective Concentrations of α-TOC and δ-TOC in the Interfacial and Oil Regions of the Emulsions

Efficient antioxidants are those whose rate of reaction with the peroxyl radicals is equal to or higher than the rate of propagation of the lipid peroxidation reaction (which corresponds to the rate of the reaction between the peroxyl radicals and the lipids). Because the rates of reactions, in addition to the rate constant, depend on the concentrations of reactants, it is crucial to determine the effective concentrations of α-TOC and δ-TOC to correctly asses their efficiency. The effective concentration of the antioxidants in the interfacial region was influenced by the stoichiometric concentration ([TOC_T_]), their distribution (%TOC_I_), and the interfacial volume (Φ_I_), Equation (9), where n_I_ and V_I_ stand for the number of moles of AO at the interfacial region and for the interfacial volume, respectively.

(9) $${(\mathrm{TOC}}_{I})=\frac{n_{I}}{V_{I}}=\frac{{[\mathrm{TOC}}_{T}{](\%\mathrm{TOC}}_{I})}{\Phi_{I}}$$

**Figure 6:**
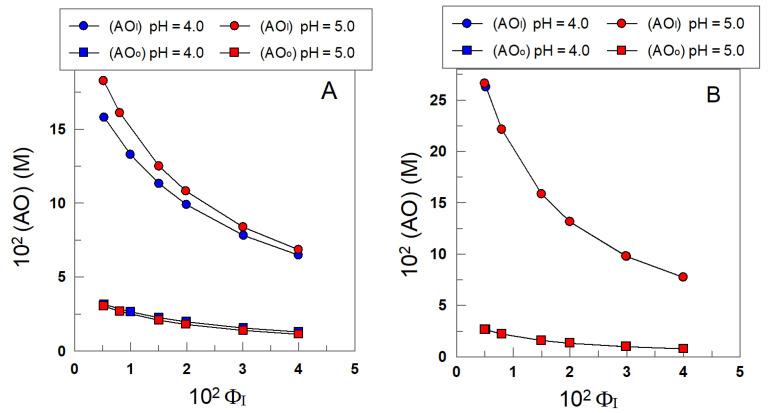

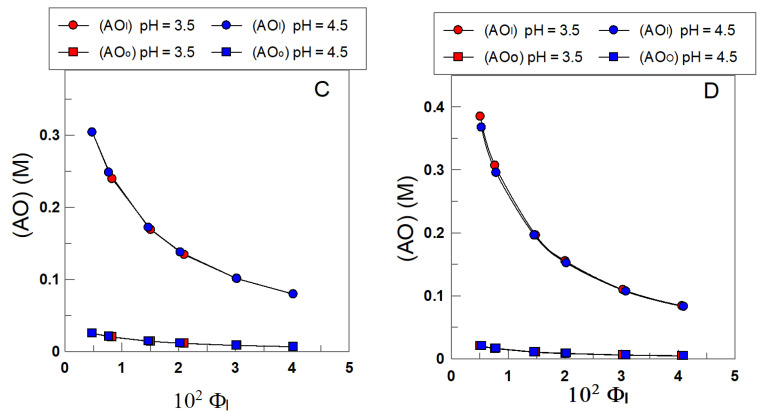
Variations of effective concentrations of α-TOC (**A**–**C**) and δ-TOC (**B**,**D**) with the surfactant volume fraction in the oil (O) and interfacial (I) regions of 1:9 (*v*:*v*) olive (**A**,**B**) and soybean (**C**,**D**) o/w emulsions. [TOC_T_] = 4 × 10^−3^ M.

At constant [TOC_T_], the effective concentration depends on the ratio between the percentage of antioxidant in the interfacial region and the interfacial volume. Both parameters increase upon increasing the amount of surfactant added to stabilize the emulsion, as shown in Figure 5A–D. However, the extent of the increase is not the same. For example, Figure 5A shows that when Φ_I_ = 0.005, %AO_I_ ≈ 20. On increasing Φ_I_ to 0.04 (that is a 10-fold increase), %AO_I_ increases to ≈70 (i.e., a 3-4-fold increase). Hence, the ratio %AO_I_/Φ_I_ decreases and, consequently, the effective interfacial concentrations decrease. Figure 6A–D show the variations in the effective concentrations in the oil and interfacial regions with the emulsifier volume fraction (Φ_I_).

The results show the remarkable observation that the effective concentration of the antioxidants in the oil region is, by far, much lower than that in the interfacial region. This may be surprising because tocopherols are lipid-soluble antioxidants. However, the interfacial region of emulsions also shows excellent solvent properties as it is a mélange of oil, surfactant, and water and concentrate reactants because its volume is much lower than that of the oil (and aqueous) regions.

At any Φ_I_ value, the effective concentrations of the antioxidants are much higher (15–96 fold) than the stoichiometric concentration ([TOC_T_] = 0.004 M). When Φ_I_ = 0.004 (the lowest value employed), the effective concentration of the antioxidants at the interfacial region follows the order α-TOC (olive) < δ-TOC (olive) < α-TOC (soybean) < δ-TOC (soybean), because the effective interfacial concentration for both tocopherols is much higher in soybean oil emulsions than in olive oil emulsion. Soybean emulsions oxidize more easily than olive oil emulsions due to the fatty acid composition of the oils. The typical composition of soybean oil is 28% oleic acid (18:1), 54% linoleic acid (18:2), 5% linolenic acid (18:3), and 13% saturated fatty acids), while that of olive oil is 76.2% oleic acid (18:1), 9.5% linoleic acid (18:2), 0.5% linolenic acid (18:3), 11.6% palmitic acid, and 2.7% stearic acid [75].

## 5. Conclusions and Final Remarks

The optimal location of the antioxidants in emulsions is in the interface of emulsions, where the inhibition reaction between the antioxidants and peroxyl radicals takes place as a result of their massive accumulation in the region [7,74,76]. The efficiency of antioxidants depends on their chemical structure, that is, on the number and location of -OH groups, double bonds, and side-chain substituents, which affects its intrinsic ability to scavenge peroxyl radicals. However, in addition to this scavenging ability, the different substituents also affect their relative hydrophobicity/hydrophilicity, resulting in a differential distribution of the antioxidants within the various regions of the emulsions [74] and its probable orientation in the interface [76].

We have recently demonstrated that the efficiency of antioxidants can be improved significantly by increasing their effective concentrations in the interfacial region, leading to significant increases in the life-time of lipid-based emulsions. Frequently, values of the oil−water partition coefficient (log *P*_W_^OCT^) are, intuitively, considered as a measure of the polarity of antioxidants. Experimentally, the partition constant is usually calculated by the shake flask method, using the corresponding oil and an aqueous phase [32,33,77]. The partition coefficient *P*_W_^O^ of a given molecule (e.g., an antioxidant) is defined as the ratio of the effective concentrations of the antioxidant in the organic and in the aqueous phase when at equilibrium. The larger value of log *P* means that the antioxidant is more hydrophobic, and it is usually considered that when log *P* > 2, the antioxidant is essentially lipid-soluble, and when log *P* ≤ −2, the solubility of the antioxidant in the aqueous phase is very high.

The evaluation of the antioxidant partitioning between different phases is an important factor for selecting antioxidants to favor their distribution toward the microenvironment that is most susceptible to oxidation. On the basis of log *P*_W_^OCT^ values, researchers sometimes make assumptions on their location and distribution in lipid-based systems [33]. However, the large differences in the solvent properties of oils compared to those of octanol make the calculated log *P*_W_^OCT^ values basically useless and frequently lead to erroneous conclusions [32]. The log *P*_W_^OIL^ values are, however, much more valuable, but still insufficient to correctly describe the distribution of antioxidants in emulsions because they are equal to the ratio of *P*_O_^I^ and *P*_w_^I^ needed to describe their distributions in emulsions. Hence, *P*_W_^OIL^ values allow one to assess whether an antioxidant is oil-insoluble or water-insoluble, but they do not allow estimations of the individual values.

Thus, it is important to develop accurate methods to estimate partition constant values in intact emulsions in order to accurately interpret (and control) the inhibition of the lipid peroxidation reaction by antioxidants. Pseudophase models are probably the most popular models for interpreting chemical reactivity under conditions where reactants are located, totally or partially, at the interfacial region of colloidal systems and where the reactants concentrate or dilute, depending on the chemical structure of the reactants and the nature and composition of the interfacial region. Therefore, the differential partitioning of reactants between the oil, water, and interfacial regions has a significant impact on the kinetics of the reaction, and the relative contributions of the medium effects (reflected in the *k*_I_ values) and the concentration effects (reflected in the partition constants) can be effectively separated and quantified, as demonstrated by the results in the present work and previously in others [7,10,31,35,74].

Pseudophase models were first introduced by Menger and Portnoy [78] for the interpretation of kinetic data of unimolecular reactions in micellar systems, but soon they were extended to bimolecular reactions, [79] photochemical reactions [43], and reactions where ions were present and the ion exchange between the bulk and interfacial regions was kinetically relevant [41,44,80]. The model was recently applied in emulsified systems, where reactants can partition among the oil, interfacial, and aqueous regions [20,21,74]. All pseudophase models assume a rapid distribution of the reactants (compared to the life time of the reaction) such that the distribution can be considered in dynamic equilibrium. The rapid distribution of reactants among the various phases or regions implies that reactants are in equilibrium with the respective activation complexes in each region such that the chemical potentials between two regions or phases are the same and, therefore, their distribution can be expressed in terms of a partition constant between both regions, which in the case of emulsions, refers to those between the oil-interfacial, *P*_O_^I^, and aqueous-interfacial, *P*_W_^I^, regions.

Pseudophase chemical methods are non-destructive and robust, and they permit examination of the parameters affecting antioxidant distribution [30,74]. Application of pseudophase models to various colloidal systems allows one, by performing an appropriate mathematical analysis, to obtain mathematical relationships relating relevant kinetic parameters, which allow one to correctly interpret chemical reactivity and quantify the relevant contributions (medium and concentration) to the rate of reactions in multiphasic systems. In the present case, the contributions are estimated from a set of kinetic data showing the variation of the measured rate constant *k_obs_* for the reaction between the chemical probe and the antioxidant at different surfactant volume fractions Φ_I_, allowing an effective separation of the medium properties of the interfacial region (reflected in the intrinsic rate constant in the interfacial region *k*_I_) from the concentration effects (reflected in the partition constants of the antioxidants). This is probably one of the most relevant outputs of the pseudophase model and what makes it very valuable in interpreting antioxidant effects in emulsions.

Finally, it is worth noting that that once the partition constants are known, one can easily calculate the distribution of an antioxidant between the oil, aqueous, and interfacial regions and, most importantly, its effective concentration in the interfacial region, which is the main reaction site between the antioxidant and the peroxyl radicals [7]. Several important conclusions can be drawn from the results: (1) at any Φ_I_, the effective interfacial concentration of δ-TOC is higher than that of α-TOC in both soybean oil and olive oil emulsions (1:9, *v*/*v*), and independent of the acidity; (2) the effective interfacial concentrations of tocopherols are much higher (15-96-fold) than the stoichiometric concentration of TOC; (3) at the lowest Φ_I_ value (Φ_I_ = 0.005), the effective interfacial concentrations of both tocopherols (δ-TOC and α-TOC) in soybean oil emulsions are higher (2-fold) than those in olive oil emulsions; and (4) predicting the interfacial concentration values of AOs in multiphasic systems and understanding the role of factors that control their location at the reaction site (interface) are crucial to controlling (and enhancing) their antioxidant efficiency in emulsions.

## Figures and Tables

**Table 1:** Values for *P*_O_^I^ of α- and δ- tocopherol (TOC) and for the rate constant in the interfacial region (*k*_I_) for the reaction between 16ArN_2_^+^ and TOC in emulsions prepared with olive and soybean oils at different acidities.

		α-TOC		δ-TOC	
Oil	pH	*P*_O_^I^	10^2^ *k*_I_ (M^−1^ s^−1)^	*P*_O_^I^	10^3^ *k*_I_ (M^−1^ s^−1)^
Soybean	3.5	12 ± 2	1.5 ± 0.4	19 ± 5	5.8 ± 0.2
	4.0	13 ± 3	4.0 ± 0.7	18 ± 4	15.9 ± 1.1
	4.5	12 ± 3	12 ± 2	18 ± 2	30.0 ± 0.4
Olive	4.0	5 ± 0	4.0 ± 0.2	10 ± 1	12.6 ± 0.3
	4.5	6 ± 1	13 ± 1.0	11 ± 2	36.5 ± 3.1
	5.0	6 ± 1	27 ± 3.0	11 ± 2	96.4 ± 5.3

## Data Availability

Not applicable.
